# {2-[(3,5-Dimethyl-2*H*-pyrrol-2-yl­idene-κ*N*)(4-nitro­phen­yl)meth­yl]-3,5-dimethyl-1*H*-pyrrol-1-ido-κ*N*}difluoridoboron

**DOI:** 10.1107/S1600536811052196

**Published:** 2011-12-10

**Authors:** Ai-Jun Cui, Jie An, Fu-An Sun, Meng Hu, Jing Qin

**Affiliations:** a Key Laboratory of Fine Petrochemical Technology, Changzhou University, Changzhou 213164, People’s Republic of China

## Abstract

In an effort to discover novel and potential boron–dipyrromethene (BODIPY) dyes, the title compound, C_19_H_18_BF_2_N_3_O_2_, was prepared from 2,4-dimethyl­pyrrole, 4-nitro­benzaldehyde and BF_3_·Et_2_O in a one-pot reaction. There are two independent mol­ecules, *A* and *B*, in the asymmetric unit in which the dihedral angles between the benzene ring and boron–dipyrromethene mean plane have significantly different values [82.71 (8)° for mol­ecule *A* and 73.16 (8)° for mol­ecule *B*]. Inter­molecular C—H⋯π inter­actions help to stabilize the crystal structure.

## Related literature

For the use of related compounds in fluorescence analysis, see: Weiner *et al.* (2001 ▶); Gabe *et al.* (2004 ▶). For related structures, see: Euler *et al.* (2002*a* ▶,*b* ▶); Cui *et al.* (2006 ▶). For the synthetic procedure, see: Kollmannsberger *et al.* (1998 ▶).
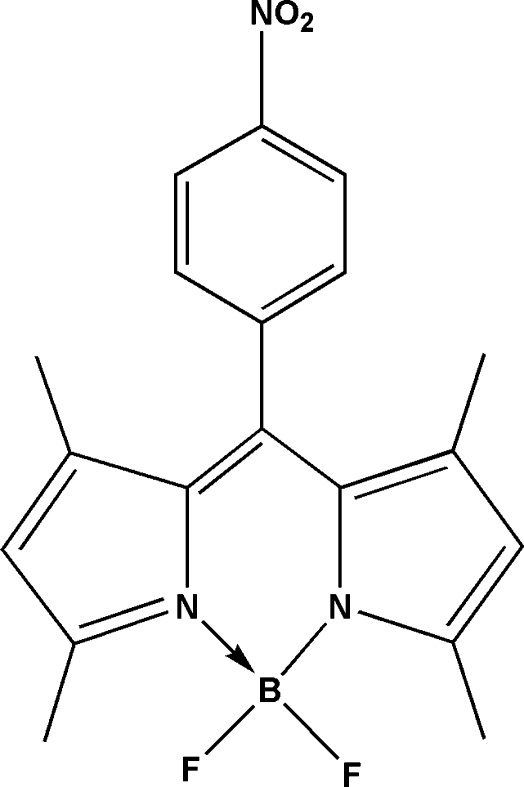


## Experimental

### 

#### Crystal data

C_19_H_18_BF_2_N_3_O_2_
*M* *_r_* = 369.17
Monoclinic, 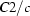

*a* = 30.5729 (6) Å
*b* = 11.8625 (2) Å
*c* = 19.8975 (5) Å
β = 96.732 (1)°
*V* = 7166.5 (3) Å^3^
*Z* = 16
Mo *K*α radiation
μ = 0.10 mm^−1^
*T* = 295 K
0.60 × 0.31 × 0.12 mm

#### Data collection

Bruker APEXII CCD area-detector diffractometer
Absorption correction: multi-scan (*SADABS*; Sheldrick, 2003 ▶) *T*_min_ = 0.961, *T*_max_ = 0.989
10802 measured reflections
6278 independent reflections
4790 reflections with *I* > 2σ(*I*)
*R*_int_ = 0.027

#### Refinement

*R*[*F* ^2^ > 2σ(*F* ^2^)] = 0.083
*wR*(*F* ^2^) = 0.193
*S* = 1.26
6278 reflections
492 parameters
H-atom parameters constrained
Δρ_max_ = 0.27 e Å^−3^
Δρ_min_ = −0.26 e Å^−3^

### 

Data collection: *APEX2* (Bruker, 2007 ▶); cell refinement: *APEX2* and *SAINT* (Bruker, 2007 ▶); data reduction: *SAINT*; program(s) used to solve structure: *SHELXTL* (Sheldrick, 2008 ▶); program(s) used to refine structure: *SHELXTL*; molecular graphics: *SHELXTL*; software used to prepare material for publication: *SHELXTL*.

## Supplementary Material

Crystal structure: contains datablock(s) I, global. DOI: 10.1107/S1600536811052196/im2338sup1.cif

Structure factors: contains datablock(s) I. DOI: 10.1107/S1600536811052196/im2338Isup2.hkl

Additional supplementary materials:  crystallographic information; 3D view; checkCIF report

## Figures and Tables

**Table 1:** Hydrogen-bond geometry (Å, °) *Cg*1 and *Cg*2 are the centroids of the N4/C26/C28/C29/C31 and N5/C21–C23/C25 rings, respectively.

*D*—H⋯*A*	*D*—H	H⋯*A*	*D*⋯*A*	*D*—H⋯*A*
C18—H18*A*⋯*Cg*1	0.93	2.93	3.784 (4)	154
C35—H35*A*⋯*Cg*2^i^	0.93	2.90	3.648 (5)	139

Symmetry code: (i) 
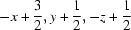
.

**Fig. 1.**
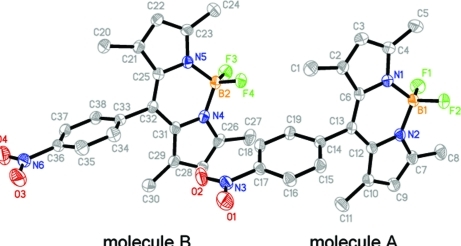
Molecular structure of the title compound with the atom-numbering scheme. Displacement ellipsoids are drawn at the 30% probability level. Hydrogen atoms are omitted for clarity.

## supplementary crystallographic information

### Comment

In the past few years, many novel boron-dipyrromethene (BODIPY) dyes were developed for fluorescence analysis (Weiner *et al.*, 2001; Gabe *et al.*, 2004) and their crystal structures were investigated at the same time (Euler *et al.*, 2002*a*,b). As part of our ongoing studies of the substituent effect on the solid-state structures of BODIPY derivatives (Cui *et al.*, 2006), we report herein the crystal strcuture of the title compound, 4,4-difluoro- 1,3,5,7-tetramethyl-8-(4'-nitrophenyl)-4-bora-3a,4a-diaza-*s*-indacene, (I).

The asymmetric unit of the title compound is shown in Fig. 1. There are two independent unique molecules [lablled *A* and *B*], in which the dihedral angles between the benzene ring and boron-dipyrromethene mean plane have significantly different values [82.71 (8)° for molecule *A*, 73.16 (8)° for molecule *B*]. In the crystal structure, there also exist intermolecular weak edge-to-face C—H···π [C18-H18A···Cg1^i^ (Cg1 = N4, C26, C28, C29, C31): H18A···Cg1, 2.93 Å, C18···Cg1, 3.784 (4) Å, i = *x*, *y*, *z*; and C35-H35A···Cg2^ii^ (Cg2 = N5, C21-C23, C25): H35A···Cg2, 2.90 Å, C35···Cg2, 3.648 (5) Å, i = -*x* + 3/2, *y* + 1/2, -*z* + 1/2 ] interactions, which help to reinforce the packing lattice.

### Experimental

Compound (I) was prepared from 2,4-dimethylpyrrole and *p*-nitrobenzaldehyde in a one-pot reaction (Kollmannsberger *et al.*, 1998). General procedure: 4.5 mmol of 2,4-dimethylpyrrole and 2 mmol of the aldehyde were dissolved in 150 ml of absolute dichloromethane under nitrogen atmosphere. One drop of trifluoroacetic acid was added and the solution was stirred at room temperature until TLC-control showed complete consumption of the aldehyde. At this point, 2 mmol dichlorodicyanobenzoquinone (DDQ) was added, and stirring was continued for 10 min followed by addition of 4 ml of triethylamine and 4 ml of boron trifluoride etherate quickly. After stirring for another 2 h, the reaction mixture was washed with water and dried, and the solvent was evaporated. The residue was chromatographed twice on a silica column (the mixture of dichloromethane and hexane as eluted solvent). Total yield: 48%. Orange crystals. ^1^H NMR (CDCl_3_): δ 1.36 (s, 6H, CH_3_), 2.57 (s, 6H, CH_3_), 6.02 (s, 2H, CH), 7.55 (d, 2H, CH, *J* = 21 Hz), 8.40 (d, 2H, CH, *J* = 22 Hz). MS (ESI), m/*z*: 368.2 [M—H]^-^. HRMS: [M—H]^-^ calculated: 368.1496, measured: 368.1472.

Red single crystals suitable for X-ray analysis were obtained by dissolving (I) (0.2 g) in a hexane/dichloromethane (15 ml, *v*:*v*: 1:3) mixture and slowly evaporating the solvent at room temperature for a period of about two weeks.

### Refinement

All H atoms bound to C atoms were assigned to calculated positions, with C—H = 0.96 Å (methyl) and 0.93 Å (aromatic), and refined using a riding model, with *U*_iso_(H)=1.2U_eq_ (C).

### Crystal data

**Table:**

C_19_H_18_BF_2_N_3_O_2_	*F*(000) = 3072
*M**_r_* = 369.17	*D*_x_ = 1.369 Mg m^−^^3^
Monoclinic, *C*2/*c*	Mo *K*α radiation, λ = 0.71073 Å
Hall symbol: -C 2yc	Cell parameters from 9250 reflections
*a* = 30.5729 (6) Å	θ = 2.9–27.5°
*b* = 11.8625 (2) Å	µ = 0.10 mm^−^^1^
*c* = 19.8975 (5) Å	*T* = 295 K
β = 96.732 (1)°	Cuboid, red
*V* = 7166.5 (3) Å^3^	0.60 × 0.31 × 0.12 mm
*Z* = 16	

### Data collection

**Table:**

Bruker APEXII CCD area-detector diffractometer	6278 independent reflections
Radiation source: fine-focus sealed tube	4790 reflections with *I* > 2σ(*I*)
graphite	*R*_int_ = 0.027
phi and ω scans	θ_max_ = 25.0°, θ_min_ = 1.3°
Absorption correction: multi-scan (*SADABS*; Sheldrick, 2003)	*h* = −30→36
*T*_min_ = 0.961, *T*_max_ = 0.989	*k* = −7→14
10802 measured reflections	*l* = −19→23

### Refinement

**Table:**

Refinement on *F*^2^	Primary atom site location: structure-invariant direct methods
Least-squares matrix: full	Secondary atom site location: difference Fourier map
*R*[*F*^2^ > 2σ(*F*^2^)] = 0.083	Hydrogen site location: inferred from neighbouring sites
*wR*(*F*^2^) = 0.193	H-atom parameters constrained
*S* = 1.26	*w* = 1/[σ^2^(*F*_o_^2^) + (0.0295*P*)^2^ + 25.3515*P*] where *P* = (*F*_o_^2^ + 2*F*_c_^2^)/3
6278 reflections	(Δ/σ)_max_ = 0.010
492 parameters	Δρ_max_ = 0.27 e Å^−^^3^
0 restraints	Δρ_min_ = −0.26 e Å^−^^3^

### Special details

**Table:**

Geometry. All e.s.d.'s (except the e.s.d. in the dihedral angle between two l.s. planes) are estimated using the full covariance matrix. The cell e.s.d.'s are taken into account individually in the estimation of e.s.d.'s in distances, angles and torsion angles; correlations between e.s.d.'s in cell parameters are only used when they are defined by crystal symmetry. An approximate (isotropic) treatment of cell e.s.d.'s is used for estimating e.s.d.'s involving l.s. planes.
Refinement. Refinement of *F*^2^ against ALL reflections. The weighted *R*-factor *wR* and goodness of fit *S* are based on *F*^2^, conventional *R*-factors *R* are based on *F*, with *F* set to zero for negative *F*^2^. The threshold expression of *F*^2^ > σ(*F*^2^) is used only for calculating *R*-factors(gt) *etc*. and is not relevant to the choice of reflections for refinement. *R*-factors based on *F*^2^ are statistically about twice as large as those based on *F*, and *R*- factors based on ALL data will be even larger.

### Fractional atomic coordinates and isotropic or equivalent isotropic displacement parameters (Å^2^)

**Table:**

	*x*	*y*	*z*	*U*_iso_*/*U*_eq_	
F2	0.40972 (7)	0.4438 (2)	0.09898 (13)	0.0572 (7)	
F3	0.70073 (9)	0.6621 (2)	0.00916 (13)	0.0657 (7)	
F4	0.66392 (7)	0.7021 (2)	0.09842 (13)	0.0587 (7)	
F1	0.44996 (9)	0.3905 (2)	0.01603 (13)	0.0635 (7)	
N1	0.48764 (10)	0.4758 (3)	0.11631 (17)	0.0435 (8)	
N2	0.43918 (10)	0.5886 (3)	0.03340 (16)	0.0409 (7)	
N5	0.74189 (10)	0.7350 (3)	0.11064 (16)	0.0416 (8)	
N3	0.60065 (13)	1.0688 (3)	0.18716 (19)	0.0551 (9)	
N4	0.69020 (10)	0.8575 (3)	0.03739 (17)	0.0456 (8)	
C32	0.75062 (12)	0.9378 (3)	0.11245 (19)	0.0390 (9)	
C12	0.46331 (12)	0.6856 (3)	0.05529 (19)	0.0412 (9)	
C13	0.49862 (11)	0.6774 (3)	0.10695 (19)	0.0375 (8)	
O2	0.63489 (11)	1.0849 (3)	0.16285 (17)	0.0702 (10)	
O1	0.58641 (13)	1.1319 (3)	0.2283 (2)	0.0891 (12)	
C6	0.51053 (12)	0.5738 (3)	0.13813 (19)	0.0404 (9)	
C31	0.71389 (12)	0.9519 (3)	0.06439 (19)	0.0434 (9)	
C33	0.77694 (12)	1.0395 (3)	0.13595 (19)	0.0414 (9)	
C14	0.52499 (12)	0.7800 (3)	0.12746 (18)	0.0387 (9)	
N6	0.85035 (16)	1.3323 (3)	0.1947 (2)	0.0703 (13)	
C18	0.59202 (13)	0.8870 (3)	0.1283 (2)	0.0460 (10)	
H18A	0.6203	0.8952	0.1160	0.055*	
C21	0.80155 (13)	0.7954 (4)	0.1816 (2)	0.0470 (10)	
C17	0.57416 (13)	0.9679 (3)	0.16588 (19)	0.0421 (9)	
C19	0.56702 (12)	0.7928 (3)	0.1091 (2)	0.0448 (9)	
H19A	0.5786	0.7371	0.0835	0.054*	
C7	0.40959 (13)	0.6181 (4)	−0.0198 (2)	0.0492 (10)	
O4	0.88462 (14)	1.3493 (3)	0.1696 (2)	0.0955 (14)	
C26	0.65831 (13)	0.8948 (4)	−0.0104 (2)	0.0539 (11)	
C23	0.76288 (13)	0.6440 (4)	0.1387 (2)	0.0486 (10)	
C38	0.81430 (13)	1.0667 (4)	0.1053 (2)	0.0478 (10)	
H38A	0.8230	1.0203	0.0716	0.057*	
C25	0.76480 (12)	0.8318 (3)	0.1355 (2)	0.0424 (9)	
C36	0.82497 (14)	1.2290 (3)	0.1748 (2)	0.0503 (10)	
C10	0.44704 (13)	0.7764 (4)	0.0132 (2)	0.0507 (10)	
B1	0.44561 (14)	0.4707 (4)	0.0653 (2)	0.0419 (10)	
B2	0.69829 (14)	0.7354 (4)	0.0628 (2)	0.0437 (11)	
C37	0.83858 (13)	1.1622 (4)	0.1247 (2)	0.0512 (11)	
H37A	0.8635	1.1808	0.1044	0.061*	
C35	0.78890 (16)	1.2029 (4)	0.2069 (2)	0.0605 (12)	
H35A	0.7808	1.2485	0.2414	0.073*	
C4	0.50634 (14)	0.3885 (4)	0.1522 (2)	0.0539 (11)	
C28	0.66037 (14)	1.0116 (4)	−0.0143 (2)	0.0604 (13)	
H28A	0.6417	1.0567	−0.0432	0.072*	
C2	0.54438 (13)	0.5435 (4)	0.1908 (2)	0.0496 (10)	
C8	0.37869 (15)	0.5364 (4)	−0.0570 (2)	0.0629 (13)	
H8A	0.3682	0.4846	−0.0255	0.094*	
H8B	0.3938	0.4955	−0.0890	0.094*	
H8C	0.3542	0.5765	−0.0806	0.094*	
C16	0.53216 (14)	0.9590 (4)	0.1841 (2)	0.0512 (10)	
H16A	0.5206	1.0158	0.2090	0.061*	
C15	0.50768 (13)	0.8643 (4)	0.1649 (2)	0.0496 (10)	
H15A	0.4794	0.8568	0.1770	0.060*	
C3	0.54139 (14)	0.4284 (4)	0.1979 (2)	0.0598 (12)	
H3A	0.5596	0.3843	0.2281	0.072*	
C29	0.69449 (13)	1.0504 (4)	0.0315 (2)	0.0512 (11)	
C20	0.83614 (15)	0.8649 (4)	0.2222 (2)	0.0633 (13)	
H20A	0.8532	0.8177	0.2545	0.095*	
H20B	0.8223	0.9233	0.2455	0.095*	
H20C	0.8551	0.8982	0.1925	0.095*	
O3	0.83572 (15)	1.3958 (3)	0.2354 (2)	0.0983 (14)	
C22	0.79973 (13)	0.6791 (4)	0.1823 (2)	0.0526 (11)	
H22A	0.8196	0.6319	0.2076	0.063*	
C34	0.76472 (15)	1.1072 (4)	0.1870 (2)	0.0547 (11)	
H34A	0.7401	1.0885	0.2081	0.066*	
C9	0.41390 (14)	0.7324 (4)	−0.0321 (2)	0.0582 (12)	
H9A	0.3971	0.7730	−0.0658	0.070*	
C11	0.46221 (17)	0.8973 (4)	0.0132 (3)	0.0782 (17)	
H11A	0.4487	0.9344	−0.0268	0.117*	
H11B	0.4937	0.8995	0.0140	0.117*	
H11C	0.4540	0.9350	0.0526	0.117*	
C27	0.62621 (15)	0.8169 (5)	−0.0502 (2)	0.0710 (14)	
H27A	0.6330	0.7403	−0.0372	0.106*	
H27B	0.6283	0.8259	−0.0976	0.106*	
H27C	0.5968	0.8346	−0.0411	0.106*	
C30	0.70618 (16)	1.1719 (4)	0.0431 (3)	0.0757 (16)	
H30A	0.6892	1.2172	0.0096	0.114*	
H30B	0.7370	1.1824	0.0397	0.114*	
H30C	0.6998	1.1939	0.0873	0.114*	
C5	0.48987 (17)	0.2703 (4)	0.1433 (3)	0.0732 (15)	
H5A	0.4657	0.2680	0.1079	0.110*	
H5B	0.4802	0.2443	0.1848	0.110*	
H5C	0.5132	0.2225	0.1315	0.110*	
C24	0.74717 (16)	0.5255 (4)	0.1245 (3)	0.0674 (13)	
H24A	0.7167	0.5199	0.1311	0.101*	
H24B	0.7642	0.4746	0.1547	0.101*	
H24C	0.7507	0.5062	0.0785	0.101*	
C1	0.57686 (15)	0.6163 (4)	0.2332 (2)	0.0654 (13)	
H1A	0.5955	0.5698	0.2640	0.098*	
H1B	0.5614	0.6690	0.2583	0.098*	
H1C	0.5945	0.6565	0.2044	0.098*	

### Atomic displacement parameters (Å^2^)

**Table:**

	*U*^11^	*U*^22^	*U*^33^	*U*^12^	*U*^13^	*U*^23^
F2	0.0406 (12)	0.0610 (16)	0.0716 (16)	−0.0079 (11)	0.0130 (11)	0.0080 (13)
F3	0.0670 (16)	0.0669 (17)	0.0618 (16)	−0.0016 (14)	0.0019 (13)	−0.0213 (14)
F4	0.0402 (13)	0.0596 (15)	0.0777 (17)	−0.0075 (11)	0.0129 (12)	0.0030 (13)
F1	0.0705 (17)	0.0545 (15)	0.0642 (16)	−0.0017 (13)	0.0020 (13)	−0.0210 (13)
N1	0.0387 (17)	0.0398 (18)	0.0515 (19)	−0.0023 (15)	0.0027 (15)	−0.0001 (15)
N2	0.0385 (17)	0.0409 (18)	0.0423 (18)	−0.0067 (14)	0.0004 (14)	−0.0022 (14)
N5	0.0382 (17)	0.0434 (19)	0.0441 (18)	−0.0041 (15)	0.0088 (14)	−0.0029 (15)
N3	0.060 (2)	0.041 (2)	0.059 (2)	−0.0081 (18)	−0.0122 (19)	0.0037 (18)
N4	0.0351 (17)	0.055 (2)	0.0464 (19)	−0.0046 (15)	0.0038 (15)	0.0011 (16)
C32	0.0324 (19)	0.046 (2)	0.039 (2)	−0.0021 (17)	0.0073 (16)	−0.0011 (17)
C12	0.040 (2)	0.041 (2)	0.043 (2)	−0.0040 (17)	0.0051 (17)	0.0022 (17)
C13	0.0322 (19)	0.041 (2)	0.040 (2)	−0.0045 (16)	0.0052 (15)	−0.0044 (17)
O2	0.065 (2)	0.069 (2)	0.075 (2)	−0.0318 (18)	0.0029 (18)	0.0075 (18)
O1	0.088 (3)	0.061 (2)	0.116 (3)	−0.009 (2)	0.005 (2)	−0.041 (2)
C6	0.036 (2)	0.042 (2)	0.044 (2)	−0.0085 (17)	0.0044 (16)	−0.0043 (17)
C31	0.037 (2)	0.050 (2)	0.044 (2)	−0.0051 (18)	0.0072 (17)	0.0024 (19)
C33	0.040 (2)	0.040 (2)	0.044 (2)	0.0010 (17)	0.0023 (17)	0.0013 (18)
C14	0.040 (2)	0.040 (2)	0.035 (2)	−0.0031 (17)	0.0004 (16)	−0.0011 (17)
N6	0.076 (3)	0.047 (2)	0.078 (3)	−0.006 (2)	−0.034 (2)	0.003 (2)
C18	0.036 (2)	0.049 (2)	0.053 (2)	−0.0078 (18)	0.0051 (18)	−0.003 (2)
C21	0.039 (2)	0.056 (3)	0.045 (2)	−0.0044 (19)	0.0024 (17)	0.006 (2)
C17	0.050 (2)	0.038 (2)	0.037 (2)	−0.0085 (18)	−0.0024 (17)	−0.0003 (17)
C19	0.037 (2)	0.047 (2)	0.051 (2)	−0.0042 (18)	0.0086 (17)	−0.0099 (19)
C7	0.044 (2)	0.055 (3)	0.047 (2)	−0.0107 (19)	−0.0028 (18)	0.001 (2)
O4	0.085 (3)	0.080 (3)	0.116 (3)	−0.035 (2)	−0.015 (3)	0.012 (2)
C26	0.040 (2)	0.076 (3)	0.045 (2)	−0.003 (2)	−0.0005 (18)	0.008 (2)
C23	0.043 (2)	0.045 (2)	0.058 (3)	0.0049 (19)	0.0096 (19)	0.005 (2)
C38	0.042 (2)	0.052 (3)	0.050 (2)	−0.0006 (19)	0.0073 (18)	−0.003 (2)
C25	0.041 (2)	0.044 (2)	0.042 (2)	−0.0020 (18)	0.0044 (17)	−0.0018 (18)
C36	0.050 (2)	0.040 (2)	0.057 (3)	−0.0040 (19)	−0.013 (2)	0.001 (2)
C10	0.045 (2)	0.048 (2)	0.058 (3)	−0.0070 (19)	−0.0022 (19)	0.008 (2)
B1	0.039 (2)	0.042 (2)	0.046 (3)	−0.007 (2)	0.0058 (19)	−0.005 (2)
B2	0.037 (2)	0.047 (3)	0.048 (3)	−0.005 (2)	0.008 (2)	−0.011 (2)
C37	0.039 (2)	0.057 (3)	0.057 (3)	−0.004 (2)	−0.0007 (19)	0.003 (2)
C35	0.070 (3)	0.053 (3)	0.057 (3)	0.000 (2)	0.003 (2)	−0.017 (2)
C4	0.049 (2)	0.042 (2)	0.070 (3)	0.0012 (19)	0.004 (2)	0.006 (2)
C28	0.045 (2)	0.076 (3)	0.059 (3)	−0.002 (2)	−0.002 (2)	0.026 (2)
C2	0.039 (2)	0.055 (3)	0.052 (2)	−0.0047 (19)	−0.0040 (18)	0.004 (2)
C8	0.056 (3)	0.070 (3)	0.060 (3)	−0.019 (2)	−0.009 (2)	−0.003 (2)
C16	0.057 (3)	0.043 (2)	0.056 (3)	−0.005 (2)	0.015 (2)	−0.009 (2)
C15	0.041 (2)	0.051 (2)	0.059 (3)	−0.0058 (19)	0.0182 (19)	−0.004 (2)
C3	0.050 (3)	0.054 (3)	0.071 (3)	0.003 (2)	−0.007 (2)	0.014 (2)
C29	0.039 (2)	0.059 (3)	0.055 (3)	−0.002 (2)	0.0039 (19)	0.014 (2)
C20	0.056 (3)	0.062 (3)	0.066 (3)	−0.008 (2)	−0.016 (2)	0.007 (2)
O3	0.117 (3)	0.058 (2)	0.111 (3)	−0.007 (2)	−0.025 (3)	−0.027 (2)
C22	0.041 (2)	0.057 (3)	0.058 (3)	0.000 (2)	0.001 (2)	0.012 (2)
C34	0.057 (3)	0.054 (3)	0.055 (3)	−0.003 (2)	0.017 (2)	−0.009 (2)
C9	0.053 (3)	0.062 (3)	0.055 (3)	−0.009 (2)	−0.012 (2)	0.016 (2)
C11	0.077 (3)	0.055 (3)	0.094 (4)	−0.019 (3)	−0.026 (3)	0.025 (3)
C27	0.055 (3)	0.094 (4)	0.060 (3)	−0.015 (3)	−0.011 (2)	−0.001 (3)
C30	0.059 (3)	0.061 (3)	0.102 (4)	−0.005 (2)	−0.009 (3)	0.028 (3)
C5	0.069 (3)	0.045 (3)	0.102 (4)	−0.001 (2)	−0.005 (3)	0.011 (3)
C24	0.066 (3)	0.047 (3)	0.088 (4)	−0.006 (2)	0.006 (3)	0.002 (3)
C1	0.060 (3)	0.069 (3)	0.061 (3)	−0.008 (2)	−0.017 (2)	0.008 (2)

### Geometric parameters (Å, °)

**Table:**

F2—B1	1.389 (5)	C23—C24	1.501 (6)
F3—B2	1.385 (5)	C38—C37	1.385 (6)
F4—B2	1.392 (5)	C38—H38A	0.9300
F1—B1	1.383 (5)	C36—C35	1.373 (6)
N1—C4	1.346 (5)	C36—C37	1.375 (6)
N1—C6	1.400 (5)	C10—C9	1.378 (6)
N1—B1	1.543 (5)	C10—C11	1.507 (6)
N2—C7	1.355 (5)	C37—H37A	0.9300
N2—C12	1.408 (5)	C35—C34	1.387 (6)
N2—B1	1.539 (6)	C35—H35A	0.9300
N5—C23	1.343 (5)	C4—C3	1.405 (6)
N5—C25	1.404 (5)	C4—C5	1.494 (6)
N5—B2	1.544 (5)	C28—C29	1.381 (6)
N3—O2	1.219 (5)	C28—H28A	0.9300
N3—O1	1.226 (5)	C2—C3	1.377 (6)
N3—C17	1.479 (5)	C2—C1	1.498 (6)
N4—C26	1.354 (5)	C8—H8A	0.9600
N4—C31	1.406 (5)	C8—H8B	0.9600
N4—B2	1.545 (6)	C8—H8C	0.9600
C32—C31	1.397 (5)	C16—C15	1.379 (6)
C32—C25	1.391 (5)	C16—H16A	0.9300
C32—C33	1.494 (5)	C15—H15A	0.9300
C12—C13	1.404 (5)	C3—H3A	0.9300
C12—C10	1.419 (5)	C29—C30	1.496 (6)
C13—C6	1.405 (5)	C20—H20A	0.9600
C13—C14	1.490 (5)	C20—H20B	0.9600
C6—C2	1.430 (5)	C20—H20C	0.9600
C31—C29	1.432 (6)	C22—H22A	0.9300
C33—C34	1.380 (6)	C34—H34A	0.9300
C33—C38	1.394 (5)	C9—H9A	0.9300
C14—C19	1.385 (5)	C11—H11A	0.9600
C14—C15	1.388 (5)	C11—H11B	0.9600
N6—O3	1.227 (6)	C11—H11C	0.9600
N6—O4	1.229 (6)	C27—H27A	0.9600
N6—C36	1.479 (6)	C27—H27B	0.9600
C18—C17	1.369 (5)	C27—H27C	0.9600
C18—C19	1.383 (5)	C30—H30A	0.9600
C18—H18A	0.9300	C30—H30B	0.9600
C21—C22	1.381 (6)	C30—H30C	0.9600
C21—C25	1.432 (5)	C5—H5A	0.9600
C21—C20	1.500 (6)	C5—H5B	0.9600
C17—C16	1.379 (6)	C5—H5C	0.9600
C19—H19A	0.9300	C24—H24A	0.9600
C7—C9	1.387 (6)	C24—H24B	0.9600
C7—C8	1.488 (6)	C24—H24C	0.9600
C26—C28	1.390 (7)	C1—H1A	0.9600
C26—C27	1.504 (6)	C1—H1B	0.9600
C23—C22	1.403 (6)	C1—H1C	0.9600
			
C4—N1—C6	108.0 (3)	C36—C37—C38	118.4 (4)
C4—N1—B1	126.0 (3)	C36—C37—H37A	120.8
C6—N1—B1	125.8 (3)	C38—C37—H37A	120.8
C7—N2—C12	108.0 (3)	C36—C35—C34	118.8 (4)
C7—N2—B1	126.7 (3)	C36—C35—H35A	120.6
C12—N2—B1	125.3 (3)	C34—C35—H35A	120.6
C23—N5—C25	108.4 (3)	N1—C4—C3	109.2 (4)
C23—N5—B2	126.4 (3)	N1—C4—C5	122.9 (4)
C25—N5—B2	125.0 (3)	C3—C4—C5	127.9 (4)
O2—N3—O1	123.9 (4)	C29—C28—C26	109.3 (4)
O2—N3—C17	118.7 (4)	C29—C28—H28A	125.4
O1—N3—C17	117.4 (4)	C26—C28—H28A	125.4
C26—N4—C31	107.7 (4)	C3—C2—C6	105.9 (4)
C26—N4—B2	127.7 (4)	C3—C2—C1	124.1 (4)
C31—N4—B2	124.5 (3)	C6—C2—C1	130.0 (4)
C31—C32—C25	121.9 (4)	C7—C8—H8A	109.5
C31—C32—C33	118.6 (3)	C7—C8—H8B	109.5
C25—C32—C33	119.4 (3)	H8A—C8—H8B	109.5
N2—C12—C13	120.0 (3)	C7—C8—H8C	109.5
N2—C12—C10	107.7 (3)	H8A—C8—H8C	109.5
C13—C12—C10	132.2 (4)	H8B—C8—H8C	109.5
C6—C13—C12	121.4 (3)	C15—C16—C17	118.7 (4)
C6—C13—C14	119.3 (3)	C15—C16—H16A	120.6
C12—C13—C14	119.3 (3)	C17—C16—H16A	120.6
C13—C6—N1	119.7 (3)	C16—C15—C14	120.5 (4)
C13—C6—C2	132.2 (4)	C16—C15—H15A	119.7
N1—C6—C2	108.1 (3)	C14—C15—H15A	119.7
C32—C31—N4	120.2 (4)	C2—C3—C4	108.8 (4)
C32—C31—C29	131.8 (4)	C2—C3—H3A	125.6
N4—C31—C29	107.9 (3)	C4—C3—H3A	125.6
C34—C33—C38	119.6 (4)	C28—C29—C31	105.7 (4)
C34—C33—C32	121.2 (4)	C28—C29—C30	124.8 (4)
C38—C33—C32	119.3 (4)	C31—C29—C30	129.5 (4)
C19—C14—C15	119.1 (4)	C21—C20—H20A	109.5
C19—C14—C13	120.4 (3)	C21—C20—H20B	109.5
C15—C14—C13	120.5 (3)	H20A—C20—H20B	109.5
O3—N6—O4	124.2 (5)	C21—C20—H20C	109.5
O3—N6—C36	117.7 (5)	H20A—C20—H20C	109.5
O4—N6—C36	118.2 (5)	H20B—C20—H20C	109.5
C17—C18—C19	118.4 (4)	C21—C22—C23	108.7 (4)
C17—C18—H18A	120.8	C21—C22—H22A	125.7
C19—C18—H18A	120.8	C23—C22—H22A	125.7
C22—C21—C25	106.2 (4)	C33—C34—C35	120.4 (4)
C22—C21—C20	124.8 (4)	C33—C34—H34A	119.8
C25—C21—C20	129.0 (4)	C35—C34—H34A	119.8
C18—C17—C16	122.2 (4)	C10—C9—C7	109.4 (4)
C18—C17—N3	118.9 (4)	C10—C9—H9A	125.3
C16—C17—N3	118.8 (4)	C7—C9—H9A	125.3
C18—C19—C14	121.1 (4)	C10—C11—H11A	109.5
C18—C19—H19A	119.5	C10—C11—H11B	109.5
C14—C19—H19A	119.5	H11A—C11—H11B	109.5
N2—C7—C9	108.8 (4)	C10—C11—H11C	109.5
N2—C7—C8	123.2 (4)	H11A—C11—H11C	109.5
C9—C7—C8	128.0 (4)	H11B—C11—H11C	109.5
N4—C26—C28	109.4 (4)	C26—C27—H27A	109.5
N4—C26—C27	122.7 (4)	C26—C27—H27B	109.5
C28—C26—C27	127.9 (4)	H27A—C27—H27B	109.5
N5—C23—C22	109.2 (4)	C26—C27—H27C	109.5
N5—C23—C24	123.2 (4)	H27A—C27—H27C	109.5
C22—C23—C24	127.6 (4)	H27B—C27—H27C	109.5
C37—C38—C33	120.5 (4)	C29—C30—H30A	109.5
C37—C38—H38A	119.8	C29—C30—H30B	109.5
C33—C38—H38A	119.8	H30A—C30—H30B	109.5
C32—C25—N5	120.0 (3)	C29—C30—H30C	109.5
C32—C25—C21	132.5 (4)	H30A—C30—H30C	109.5
N5—C25—C21	107.5 (3)	H30B—C30—H30C	109.5
C35—C36—C37	122.3 (4)	C4—C5—H5A	109.5
C35—C36—N6	119.2 (4)	C4—C5—H5B	109.5
C37—C36—N6	118.5 (4)	H5A—C5—H5B	109.5
C9—C10—C12	106.2 (4)	C4—C5—H5C	109.5
C9—C10—C11	124.3 (4)	H5A—C5—H5C	109.5
C12—C10—C11	129.5 (4)	H5B—C5—H5C	109.5
F2—B1—F1	109.4 (3)	C23—C24—H24A	109.5
F2—B1—N2	109.7 (3)	C23—C24—H24B	109.5
F1—B1—N2	110.6 (3)	H24A—C24—H24B	109.5
F2—B1—N1	109.6 (3)	C23—C24—H24C	109.5
F1—B1—N1	110.6 (3)	H24A—C24—H24C	109.5
N2—B1—N1	106.9 (3)	H24B—C24—H24C	109.5
F3—B2—F4	109.1 (3)	C2—C1—H1A	109.5
F3—B2—N5	110.6 (3)	C2—C1—H1B	109.5
F4—B2—N5	109.5 (3)	H1A—C1—H1B	109.5
F3—B2—N4	110.9 (4)	C2—C1—H1C	109.5
F4—B2—N4	109.3 (3)	H1A—C1—H1C	109.5
N5—B2—N4	107.3 (3)	H1B—C1—H1C	109.5
			
C7—N2—C12—C13	−175.3 (4)	O4—N6—C36—C37	−5.9 (6)
B1—N2—C12—C13	5.0 (6)	N2—C12—C10—C9	0.2 (5)
C7—N2—C12—C10	0.2 (4)	C13—C12—C10—C9	175.0 (4)
B1—N2—C12—C10	−179.4 (4)	N2—C12—C10—C11	−177.7 (5)
N2—C12—C13—C6	−0.4 (6)	C13—C12—C10—C11	−2.9 (8)
C10—C12—C13—C6	−174.7 (4)	C7—N2—B1—F2	−70.0 (5)
N2—C12—C13—C14	177.4 (3)	C12—N2—B1—F2	109.6 (4)
C10—C12—C13—C14	3.1 (7)	C7—N2—B1—F1	50.8 (5)
C12—C13—C6—N1	1.7 (6)	C12—N2—B1—F1	−129.7 (4)
C14—C13—C6—N1	−176.1 (3)	C7—N2—B1—N1	171.2 (4)
C12—C13—C6—C2	−179.9 (4)	C12—N2—B1—N1	−9.2 (5)
C14—C13—C6—C2	2.3 (7)	C4—N1—B1—F2	65.3 (5)
C4—N1—C6—C13	177.7 (4)	C6—N1—B1—F2	−108.3 (4)
B1—N1—C6—C13	−7.8 (6)	C4—N1—B1—F1	−55.4 (5)
C4—N1—C6—C2	−1.0 (5)	C6—N1—B1—F1	131.1 (4)
B1—N1—C6—C2	173.5 (4)	C4—N1—B1—N2	−175.8 (4)
C25—C32—C31—N4	−0.5 (6)	C6—N1—B1—N2	10.6 (5)
C33—C32—C31—N4	175.4 (3)	C23—N5—B2—F3	−52.3 (5)
C25—C32—C31—C29	−177.0 (4)	C25—N5—B2—F3	131.9 (4)
C33—C32—C31—C29	−1.2 (6)	C23—N5—B2—F4	67.9 (5)
C26—N4—C31—C32	−176.4 (4)	C25—N5—B2—F4	−107.8 (4)
B2—N4—C31—C32	7.4 (6)	C23—N5—B2—N4	−173.5 (4)
C26—N4—C31—C29	1.0 (4)	C25—N5—B2—N4	10.8 (5)
B2—N4—C31—C29	−175.3 (4)	C26—N4—B2—F3	52.0 (5)
C31—C32—C33—C34	84.0 (5)	C31—N4—B2—F3	−132.5 (4)
C25—C32—C33—C34	−100.1 (5)	C26—N4—B2—F4	−68.3 (5)
C31—C32—C33—C38	−95.5 (4)	C31—N4—B2—F4	107.2 (4)
C25—C32—C33—C38	80.4 (5)	C26—N4—B2—N5	173.0 (4)
C6—C13—C14—C19	71.7 (5)	C31—N4—B2—N5	−11.5 (5)
C12—C13—C14—C19	−106.2 (4)	C35—C36—C37—C38	1.4 (6)
C6—C13—C14—C15	−108.7 (4)	N6—C36—C37—C38	−178.8 (4)
C12—C13—C14—C15	73.4 (5)	C33—C38—C37—C36	0.1 (6)
C19—C18—C17—C16	−0.9 (6)	C37—C36—C35—C34	−1.6 (7)
C19—C18—C17—N3	179.6 (4)	N6—C36—C35—C34	178.6 (4)
O2—N3—C17—C18	10.3 (5)	C6—N1—C4—C3	0.3 (5)
O1—N3—C17—C18	−170.1 (4)	B1—N1—C4—C3	−174.1 (4)
O2—N3—C17—C16	−169.2 (4)	C6—N1—C4—C5	178.8 (4)
O1—N3—C17—C16	10.4 (6)	B1—N1—C4—C5	4.3 (7)
C17—C18—C19—C14	−0.2 (6)	N4—C26—C28—C29	0.5 (6)
C15—C14—C19—C18	1.0 (6)	C27—C26—C28—C29	179.3 (4)
C13—C14—C19—C18	−179.4 (4)	C13—C6—C2—C3	−177.2 (4)
C12—N2—C7—C9	−0.6 (5)	N1—C6—C2—C3	1.3 (5)
B1—N2—C7—C9	179.1 (4)	C13—C6—C2—C1	4.2 (8)
C12—N2—C7—C8	178.9 (4)	N1—C6—C2—C1	−177.3 (4)
B1—N2—C7—C8	−1.4 (6)	C18—C17—C16—C15	1.2 (6)
C31—N4—C26—C28	−0.9 (5)	N3—C17—C16—C15	−179.3 (4)
B2—N4—C26—C28	175.2 (4)	C17—C16—C15—C14	−0.4 (6)
C31—N4—C26—C27	−179.8 (4)	C19—C14—C15—C16	−0.7 (6)
B2—N4—C26—C27	−3.7 (7)	C13—C14—C15—C16	179.7 (4)
C25—N5—C23—C22	0.1 (5)	C6—C2—C3—C4	−1.1 (5)
B2—N5—C23—C22	−176.2 (4)	C1—C2—C3—C4	177.6 (4)
C25—N5—C23—C24	179.0 (4)	N1—C4—C3—C2	0.5 (6)
B2—N5—C23—C24	2.7 (6)	C5—C4—C3—C2	−177.9 (5)
C34—C33—C38—C37	−1.4 (6)	C26—C28—C29—C31	0.1 (5)
C32—C33—C38—C37	178.1 (4)	C26—C28—C29—C30	−178.4 (5)
C31—C32—C25—N5	−0.3 (6)	C32—C31—C29—C28	176.2 (4)
C33—C32—C25—N5	−176.1 (3)	N4—C31—C29—C28	−0.7 (5)
C31—C32—C25—C21	177.4 (4)	C32—C31—C29—C30	−5.3 (8)
C33—C32—C25—C21	1.7 (7)	N4—C31—C29—C30	177.8 (5)
C23—N5—C25—C32	177.8 (4)	C25—C21—C22—C23	−0.6 (5)
B2—N5—C25—C32	−5.8 (6)	C20—C21—C22—C23	179.6 (4)
C23—N5—C25—C21	−0.5 (4)	N5—C23—C22—C21	0.3 (5)
B2—N5—C25—C21	175.9 (3)	C24—C23—C22—C21	−178.6 (4)
C22—C21—C25—C32	−177.4 (4)	C38—C33—C34—C35	1.2 (7)
C20—C21—C25—C32	2.5 (8)	C32—C33—C34—C35	−178.3 (4)
C22—C21—C25—N5	0.6 (5)	C36—C35—C34—C33	0.3 (7)
C20—C21—C25—N5	−179.5 (4)	C12—C10—C9—C7	−0.5 (5)
O3—N6—C36—C35	−6.1 (6)	C11—C10—C9—C7	177.5 (5)
O4—N6—C36—C35	173.9 (4)	N2—C7—C9—C10	0.7 (5)
O3—N6—C36—C37	174.1 (4)	C8—C7—C9—C10	−178.8 (4)

### Hydrogen-bond geometry (Å, °)

**Table:**

Cg1 and Cg2 are the centroids of the N4/C26/C28/C29/C31 and N5/C21–C23/C25 rings, respectively.

**Table:**

*D*—H···*A*	*D*—H	H···*A*	*D*···*A*	*D*—H···*A*
C18—H18A···Cg1	0.93	2.93	3.784 (4)	154
C35—H35A···Cg2^i^	0.93	2.90	3.648 (5)	139

Symmetry codes: (i) −*x*+3/2, *y*+1/2, −*z*+1/2.
